# Recent insights into the biological and pharmacological activity of lycopene

**DOI:** 10.17179/excli2022-4714

**Published:** 2022-02-16

**Authors:** Jae Kwang Kim, Sang Un Park

**Affiliations:** 1 Division of Life Sciences and Bio?Resource and Environmental Center, College of Life Sciences and Bioengineering, Incheon National University, Incheon 22012, Korea; 2 Department of Crop Science, Chungnam National University, 99 Daehak-ro, Yuseong-gu, Daejeon 34134, Korea; 3 Department of Smart Agriculture Systems, Chungnam National University, 99 Daehak-ro, Yuseong-gu, Daejeon 34134, Korea

## ⁯

Lycopene is a C40 tetraterpene that is composed of eight isoprene units joined by regular head to tail bindings, except in the middle of the molecule, where tail to tail binding forms an asymmetric structure (Arballo et al., 2021[4]). It is a naturally occurring lipophilic red-colored carotenoid pigment found in many red-colored fruits and vegetables, especially tomatoes (Story et al., 2010[67]; Grabowska et al., 2019[31]; Arballo et al., 2021[4]; Sathasivam et al., 2021[64]). Lycopene cannot be synthesized in the human body and must be obtained from dietary foods (Woodside et al., 2015[78]; Imran et al., 2020[36]). This dietary lycopene is stored in adrenals, liver, and the prostate and in lower concentrations in other parts of the body, such as the brain and skin (Moran et al., 2013[49]; Imran et al., 2020[36]).

Lycopene has a potent antioxidant activity and is important for scavenging reactive oxygen species (ROS) (Imran et al., 2020[36]). Its ability to remove atomic oxygen is ten and two times higher than that of α-tocopherol and β-carotene, respectively (Przybylska, 2020[56]). Because of its numerous health benefits, the interest in lycopene consumption has increased. Additionally, lycopene is beneficial for the prevention and treatment of various diseases, including neurodegenerative diseases like Parkinson's and Alzheimer's (Cho et al., 2018[13]; Joshi et al., 2020[39]; Przybylska, 2020[56]). Administration of high doses of lycopene and its products can cure chronic conditions like cardiovascular diseases, cancer (lung and prostate cancers), non-alcoholic fatty liver disease, neurological disorders, oxidative stress, inflammatory pathways, and male infertility (Shen et al., 2007[65]; Chung et al., 2012[16]; Ip et al., 2015[37]; Abar et al., 2016[1]). In addition, lycopene not only inhibits the proliferation of neoplastic cells but also stimulates their apoptosis and inhibits metastasis (Przybylska, 2020[56]). However, the mechanism by which lycopene executes its bioactivity remains unknown (Arballo et al., 2021[4]). Herein, we compiled the most recent findings regarding the biological and pharmacological activities of lycopene (Table 1(Tab. 1); References in Table 1: Albrahim and Alonazi, 2021[2]; Arakeri et al., 2020[3]; Asgary et al., 2021[5]; Babaei et al., 2021[6]; Bedir et al., 2021[7]; Cao et al., 2021[8]; Celik et al., 2020[9]; Chen et al., 2019[10], 2021[11]; Cheng et al., 2020[12]; Choi and Kim, 2020[15]; Choi et al., 2021[14]; de Barros Elias et al., 2019[17]; Dobrzyńska and Gajowik, 2020[18]; Dobrzyńska et al., 2019[19]; Duan et al., 2019[20]; Eita et al., 2021[21]; El Morsy and Ahmed, 2020[22]; Elgawish et al., 2020[23]; Eze et al., 2019[24]; Fachinello et al., 2020[25]; Fallah et al., 2021[26]; Fan et al., 2019[27]; Farouk et al., 2021[28]; Fu et al., 2020[29]; Gao et al., 2019[30]; Greish et al., 2021[32]; Hashimoto et al., 2020[33]; Huang et al., 2019[34]; İkiz et al., 2021[35]; Jesuz et al., 2019[38]; Kang et al., 2021[40]; Kim et al., 2019[41]; Lee et al., 2021[42]; Leh et al., 2021[43]; Li et al., 2021[44]; Liu et al., 2021[45][46]; Ma et al., 2019[47]; Malekiyan et al., 2019[48]; Motallebnejad et al., 2020[50]; Mustra Rakic et al., 2021; Ni et al., 2020[51]; Ning et al., 2021[52]; Ou et al., 2020[53]; Park et al., 2020[54]; Polat et al., 2021[55]; Rajput et al., 2021[57]; Rasheed et al., 2020[59]; Residiwati et al., 2021[60]; Rocha et al., 2021[61]; Róvero Costa et al., 2019[62]; Sarker et al., 2021[63]; Soares et al., 2019[66]; Sun et al., 2021[68]; Tao et al., 2021[69]; Taskiran and Tastemur, 2021[70]; Turkler et al., 2020[71]; Ugbaja et al., 2021[72]; Ugwor et al., 2021[73]; Wadie et al., 2021[74]; Wang et al., 2020[77], 2021[75], 2022[76]; Xu et al., 2019[79]; Xue et al., 2021[80]; Yilmaz et al., 2020[81]; Zhang et al., 2020[82]; Zhao et al., 2020[85], 2021[83][84]; Zhu et al., 2020[86]).

## Declaration

### Acknowledgments

This work was supported by the Incheon National University Research Concentration Professors Grant in 2021.

### Conflict of interest

The authors declare no conflict of interest.

## Figures and Tables

**Table 1:**
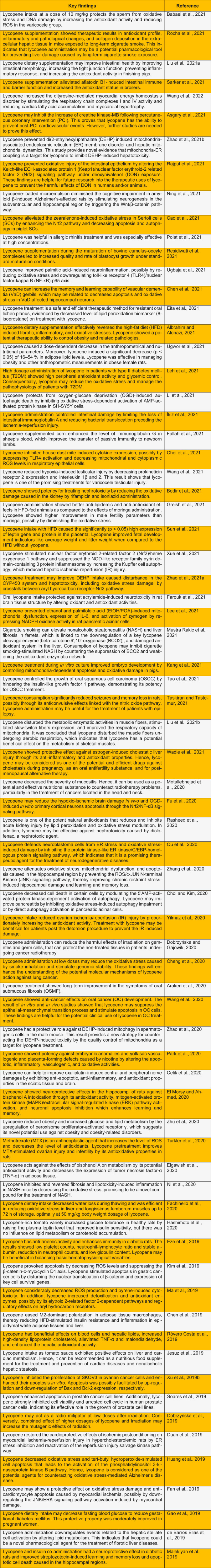
Recent studies on the biological and pharmacological activities of lycopene
